# Fireflies in Art: Emphasis on Japanese Woodblock Prints from the Edo, Meiji, and Taishō Periods

**DOI:** 10.3390/insects13090775

**Published:** 2022-08-27

**Authors:** Deirdre A. Prischmann-Voldseth

**Affiliations:** Entomology Department, North Dakota State University, Fargo, ND 58108-6050, USA; deirdre.prischmann@ndsu.edu; Tel.: +1-(701)231-9805

**Keywords:** Lampyridae, cultural entomology, *ukiyo-e*, conservation, eco-art

## Abstract

**Simple Summary:**

Fireflies are beetles (Coleoptera: Lampyridae) famous for their bioluminescence. This study examined artistic representations of fireflies and depictions of how people interacted with these insects in Japan from a historical perspective. Visual information from the artwork was summarized, highlighting themes and connections to firefly biology and cultural entomology. Multiple artists were represented, including several renowned masters, and the artwork highlights the complex interactions between fireflies and humans. Analyzing artwork can enhance awareness of the historical and cultural significance of insects and may help with conservation efforts.

**Abstract:**

Examining how insects are represented in artwork can provide insight into people’s perceptions and attitudes towards arthropods, as well as document human–insect interactions and how they change through time. Fireflies are well-known bioluminescent beetles (Coleoptera: Lampyridae) of great cultural significance, especially in Japan. A selection of online museum collections, art databases, and dealer websites were used to find artwork featuring fireflies, with an emphasis on Japanese *ukiyo-e* wood block prints from the Edo, Meiji, and Taishō time periods (1600–1926). Quotes from early twentieth century texts were used to provide additional historical context. Over 90 different artists created artwork featuring fireflies, including several renowned masters. Artists depicted adult fireflies in a variety of ways (e.g., relatively accurately, more generalized, symbolic or abstract, yellowish dots) in the absence and presence of people. Most images were set outdoors during the evening near water, and primarily featured women and children, groups of women, and large parties catching fireflies or observing caged fireflies. ‘Beauties’, *geisha*, courtesans, *kabuki* actors, and insect vendors were also common subjects. Various types of collecting tools and a diversity of cages were featured, as well as insect vendors. The artwork highlights the complex connections between fireflies and humans. Insect-related art can contribute to education and conservation efforts, particularly for dynamic insects such as fireflies that are facing global population declines.

## 1. Introduction

Art is a means of expression and a valuable communication tool. Examining artistic representations of insects and their relatives can provide insight into people’s perceptions and attitudes towards arthropods, as well as document human–insect interactions and how they change through time [1,2,3,4,5,6]. Insect-related artwork can also contribute to educational efforts [7,8], and stimulate discussion about modern societal concerns, e.g., impacts of anthropogenic activities on the environment [9] and conservation of culturally important insects, such as fireflies [10,11].

Fireflies are beetles (Coleoptera: Lampyridae) famous for their bioluminescence. There are over 2500 species of lampyrids [12]. They have a broad geographic distribution and all produce light at some point during their life cycle, although not all adult fireflies bioluminesce [13]. Researchers have used mating behavior signals to group North American fireflies into three categories: diurnal species that rely on pheromones (dark fireflies), larviform females that glow and alate males that do not (glowworm fireflies), and alate females and males that both flash (lighteningbugs) [13,14]. People are likely the most familiar with the latter (e.g., *Photinus*, *Photuris*, *Luciola* spp.), where crepuscular or nocturnal adult males and females use light for communication and mating, or in the case of predatory fireflies to attract prey [13,15,16,17]. Adult fireflies are chemically protected and can engage in reflexive bleeding as a defense mechanism [18,19]. Fireflies spend most of their lives as immatures, with non-feeding or predatory adults only living a few weeks [20]. Immatures are found most often in damp habitats, and depending on the species, larvae can be aquatic, semi-aquatic, terrestrial, arboreal, or subterranean [21]. They typically feed on gastropods such as snails [20], and Fabre [22] provides a vivid account of an attack by what is likely a glowworm firefly. Species that are important in Japanese culture are dependent on water (e.g., creeks, rice paddy fields) as immatures [23].

Fireflies are known by many monikers, e.g., botaru, fuogola, glow-worms, glühwürmers, hotaru, lampyris, leuchtkafers, liegthmugh, lighting-bugs, luciernega, lucioles, mouches de feu, vers-luisants, and shine-worms [24,25], and Harvey [26] lists many more historical names. Fireflies appear in multiple ancient texts [26] and are of great cultural significance in Japan, both historically and currently [23,27,28,29,30,31,32]. Although at least 50 species have been recorded from Japan [33], three species are the most well-known, likely due to their bioluminescence, broad distribution within the country, and proximity to humans: Genji-botaru or Minamoto-Firefly (*Luciola cruciata* Motschulsky), Heike-botaru or Taira-Firefly (*Aquatica lateralis* Motschulsky) and Hime-botaru (*Luciola parvula* Kiesenwetter) [25,33,34]. *Luciola cruciata* is a designated national natural treasure, and people are highly interested in its conservation, especially as the larval stage is aquatic and vulnerable to water pollution [28,33].

Due to their cultural importance, fireflies were a common subject in Japanese artwork. The creation of paintings and woodblock prints known as *ukiyo-e* or “pictures of the floating world” that showed daily life, entertainment, or leisure activities were common in the seventeenth to the twentieth centuries, and mass-produced color woodblock printing became a major commercial enterprise, especially around Edo (i.e., Toyko) [35,36]. The artists who designed the images typically were credited with their creation, although the process also involved engravers who carved the wood blocks, printers who inked the designs and transferred them to paper, and publishers who provided funding [35,36]. Woodblock printing evolved as time passed, with black ink images (*sumizuri-e*) followed by hand colored and printed images with a pinkish color (*beni-e* and *benizuri-e*), eventually giving way to multicolored ‘brocade’ pictures (*nishiki-e*) [35]. Multi-sheet images (e.g., diptychs, triptychs, etc.) also become more common over time, especially in the latter half of the eighteenth century [36].

This study examined artistic representations of fireflies and depictions of how people interacted with these insects in Japan from a historical perspective. The information within the images was summarized, highlighting themes and connections to firefly biology and cultural entomology.

## 2. Materials and Methods

Several sources were used to find relevant artwork, including 15 open access museum collections, art museums connected to the Google Arts & Culture website (artsandculture.google.com), the United States Library of Congress (www.loc.gov), the Nagaski University Library collection of ‘Japanese Old Photographs in Bakumatsu-Meiji Period’ (http://oldphoto.lb.nagasaki-u.ac.jp/top/en_top.php), an image database supporting research on Japanese woodblock prints (ukiyo-e.org), and art dealer websites (fujiarts.com), (sothebys.com). Search terms were: ‘firefly’, ‘fireflies’, ‘Hotaru’, ‘Genji’, ‘insect’, ‘insect cage’, or ‘cage’. Websites for images referenced in this study were accessed multiple times from 4 January 2022 to 22 August 2022 and are listed in Table A1, and each work was given a unique identification number (i.e., a T-number).

Artwork from multiple time periods was examined: Edo (Tokugawa) period (1600–1868), Meiji period (1868–1912), Taishō period (1912–1926), and Shōwa period (1926–1989) [37,38]. The emphasis was on Japanese wood block prints from the first three time periods, and quotes from early 20th century texts were used to provide additional historical context.

## 3. Results

**Figure 1:**
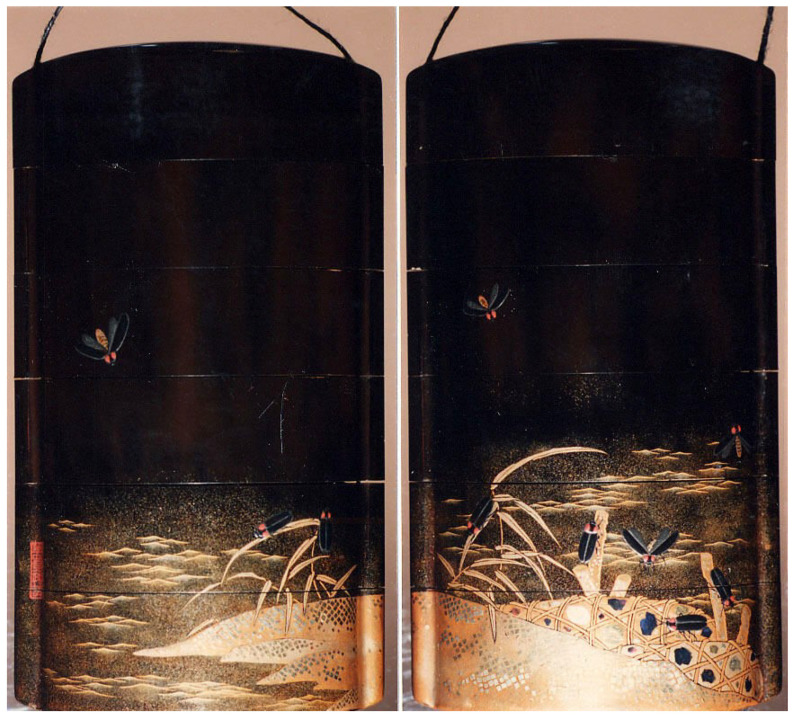
(T100). Two sides of Case (Inrō) with Design of Fireflies in Flight and Climbing on Stone Baskets and Reeds at the Shore. Noneteenth C. Unknown artist (Japanese). Lacquer, roiro, gold and coloured hiramakie, togidashi, nashiji, kirigane; Interior: nashiji and fundame. H.O. Havemeyer Collection, Bequest of Mrs. H.O. Havemeyer, 1929. Accession Number: 29.100.913. The Metropolian Museum of Art, New York, NY, USA, www.metmuseum.org (accessed on 4 January 2022). Open access image, CC0.

In total, over 200 works of art were assessed in this study (Table A1), although there are undoubtedly more in other museums and collections. Over 94 different artists were represented, including several renowned masters, e.g., Katsushika Hokusai, creator of the iconic ‘The Great Wave’, Utagawa Kunisada I, Utagawa Kuniyoshi, and Kitagawa Utamaro [36,39]. Multiple prints of the same piece were often found, typically at different museums or art websites. In addition to woodblock prints, paintings, lithographs, and photographs, firefly or insect cage motifs adorned several types of objects, including: boxes (T163, T173, T176-177, T181), dishes (T46, T175), pipe cases (T103), netsuke (miniature sculptures; T104-105), inrō (case for small objects; T61, T154, T191; T100, Figure 1), kozuka (small knife; T27, T147), and robes (T178-179).

### 3.1. Focus on Fireflies

Artists depicted adult fireflies in the absence or presence of people, with the apparent lack of immatures perhaps related to their more cryptic nature. Representations of adults were diverse, whether free-ranging or contained within cages, and ranged from realistic-looking insects to yellow-colored or golden dots. Research indicates flashes of crepuscular firefly species are yellower than the greener flashes emitted by nocturnal fireflies [40]. ‘Fireflies at Ochanomizu’ by Kiyochika (T49) is a good example of where the fireflies’ light was greenish or chartreuse, rather than golden yellow. Some fireflies appeared to be generalized insects (e.g., T54) or resembled butterflies (e.g., T144-146, T175) or were more abstract, such as a blotch or the letter ‘X’ (T171). However, in other pieces the insects were clearly fireflies, with elongate bodies, well defined elytra, segmented abdomens, pink coloration on the pronotum, and the distal end of the abdomen greenish-yellow or depicted as glowing. Table A1 provides information on how fireflies were represented in the artwork (i.e., letter codes after the title). Of pieces clearly related to fireflies where the insects could be seen (with multiple sheets of a triptych counted as one image), 22% of artworks had relatively accurate depictions of fireflies, 32% had less accurate and more generalized fireflies, 26% had more symbolic or abstract fireflies, and 20% had fireflies represented by yellowish or greenish dots. Additionally, 47% had some indication of firefly bioluminescence (e.g., yellowish or greenish abdomen or dots at the end of the abdomen. Artwork by certain artists, such as Sōzan and Zeshin, had more accurate representations of fireflies. In general, the more accurate depictions of fireflies tended to be on artwork lacking people or on objects. With regard to the latter, fireflies on two carved netsuke (T104-105) had a rounder body and reddish pronotum reminiscent of a less common diurnal firefly from Japan, *Cyphonocerus ruficollis* Kiesenwetter [17,41,42]. Images of fireflies interacting with other animals were rare, perhaps because fireflies are chemically protected [18,19], although one print showed fireflies above what looks like an interested dragonfly naiad (T167), and another showed a firefly trapped in a spider’s web (T170). Lafcadio Hearn (b.1850–d.1904), an author who wrote extensively about Japan [43] reported, “*[…] the firefly has a very bitter taste, and birds appear to find it unpalatable. (Frogs […] do not mind the bad taste: they fill their cold bellies with fireflies till the light shines through them […]).*” [34] (p. 138).

**Figure 2:**
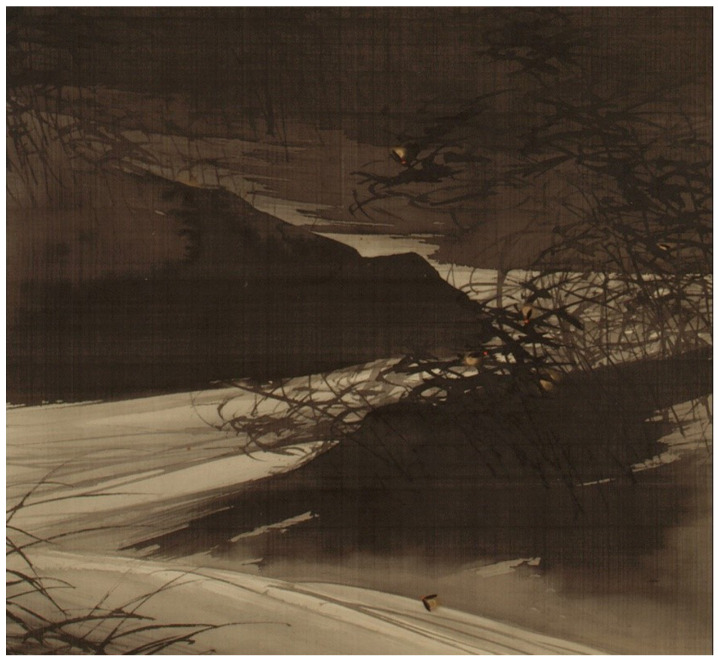
(T127). Fireflies Over the Uji River by Moonlight (detail). Meiji period (1868–1912). Suzuki Shōnen (Japanese, 1849–1918). Painting, hanging scroll; ink, color, and gold on silk. Purchase, Gift of Mrs. Russell Sage, by exchange, 1979. Accession Number: 1979.72. The Metropolian Museum of Art, New York, USA, www.metmuseum.org (accessed 4 January 2022). Open access image, CC0.

Two-dimensional artwork focused solely on the insects fell into two broad categories. Several pieces, including multiple works by Zeshin, had a few non-glowing fireflies flying or at rest, usually outdoors in the daytime surrounded by white space (T115-116, T161, T166, T168-169, T199-200, T203) or in still-lifes with flat uchiwa fans, decorative trays, or lanterns (T174, T198, T202). A few prints showed fireflies on plants with greenish-gray or dark gray backgrounds (T183, T197). A second group of paintings and prints depicted fireflies—often dozens of them—at night near water. These night scenes had muted gray or black color palettes, which highlighted the fireflies’ reddish body parts and luminescence, and included woodblock prints by Kōgyō (T59), Koson (T65), and Sōzan (T148-149) and paintings by Shōnen (T126-127, Figure 2) and Bunrin (T4-8). Bunrin often depicted fireflies [44], and the gallery label for his ‘River Landscape with Fireflies’ (T4) reads, “*[…] tiny golden flashes of fireflies along a riverbank evoke the charms of summer. Tufts of bamboo, willow trees, and cascading waters convey the cool nocturnal atmosphere. By incorporating naturalistic effects such as the brushwork and ink tones that capture the volume of rocks and water, Bunrin created a sensation of rushing waters and cooling nighttime breezes.*”

Some night scenes had human elements, such as buildings (T151, T155) and indistinct figures on boats (T49) or behind window shades (T50), although the fireflies were the primary element of interest. Kiyochika’s print ‘Koromogawa River at Tennōji-shita’, (T50) was reproduced in a book based on the experiences of an English woman who lived in Japan for several years, who recounts of her journey to Ikao, “*Suddenly, in a lull of the rain, I saw a great white star moving slowly down towards me out of the sky. Only when it floated close to my eyes did I discover that it was the very patriarch of all the fireflies […].*” [45] (p. 27). Several images had silhouetted figures, including work by Gekkō (T24), Hiroaki (T131), Shōtei (T130, T134), Shōun (T136), Toshihide (T156), and Toshikata (T157), and one had the background landscape and plants in silhouette while the people in the foreground were in vibrant color (T88).

**Figure 3:**
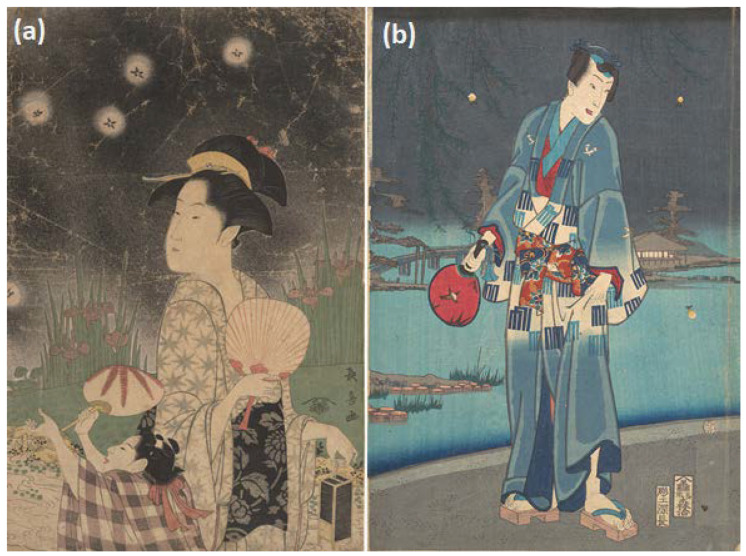
The Metropolian Museum of Art, New York, USA, www.metmuseum.org (accessed on 4 January 2022). Open access images, CC0. (**a**) (T16). Woman and Child Catching Fireflies. ca. 1793. Eishōsai Chōki (Japanese, active late 18th–early 19th C.). Woodblock print; ink and color on paper. H.O. Havemeyer Collection, Bequest of Mrs. H.O. Havemeyer, 1929. Accession Number: JP1739. (**b**) (T67). Modern Genji—Firefly Viewing (Imayō genji shiken hotaru asobi). 1961. Toyohara Kunichika, (Japanese, 1835–1900). Central sheet of a triptych; woodblock print, ink and color on paper. Museum Accession. Accession Number: JP1093.1.

Artists used different strategies to convey nocturnal or twilight settings. Some prints had a solid black background (e.g., Chōki T16, Figure 3a), a grayish (e.g., T10, T73-74, T79, T129, T157) or blue sky (e.g., Kunichika T67, Figure 3b). Many images had a lighter background with a dark streak at the top (e.g., T20, T44, T78, T90, T92, T112), and one had an orange horizon akin to a sunset (T141). However, many images of people watching or collecting fireflies had a light background without any indication of darkness (e.g., T42, T140, T144-146, T158), some of which were *benizuri-e* style prints (T54, T182).

### 3.2. Settings

Watching and hunting fireflies for entertainment has been a popular custom in Japan for centuries, and Hearn [34] (p. 149) indicated that, “*anciently it was an aristocratic amusement; and great nobles used to give firefly-hunting parties,—hotaru-gari*”. Many places were famous for their fireflies, such as the Hotaru-Dani (Valley of the Fireflies) near Ishiyama, the lake of Ōmi, and Uji in Yamashiro Province [34,46,47]. An early travel guide [46] (p. 552) based on [34] talked about the Battle of the Fireflies (*Hotaru-Kassen*) near Uji that happened annually around June 10th at midnight. “*[…] thousands of persons come hither from Kyōto (tram-cars), Ōsaka, Kobe, and nearby cities to witness the brilliant struggle. […] The battle […] occurs on the river between Uji and Fushimi, about 1½ hrs. boat ride from the former place […]. The uncounted millions of sparkling insects produce a scene of bewildering beauty as they wheel and circle […], and the scores of illuminated boats on which there are dancing and singing, geisha, music, and jollity, add to the charm. When the fireflies have assembled in force myriads dart from either bank and meet and cling above the water. At moments they so swarm together as to form what appears to the eye like a luminous cloud, or like a great ball of sparks. […] After the Hotaru-Kassen is done, the river is covered with the still sparkling bodies of the drifting insects. Then the natives refer poetically to the stream as the ‘Milky Way;, the ‘River of Heaven’, etc.*”

Some pieces of art mentioned specific locations, many of which were renowned for their fireflies. Two of Shōnen’s hanging scroll paintings depicted fireflies over the Uji River (T126-127), and Tokuriki had a piece featuring the Uji River in his ’15 Views of Kyoto’ series (T155). Kunisada I created a print of women catching fireflies by the Uji River (T88), a print listed as, ‘Catching Fireflies at Sekiya’, (T71), which is a village by the Sumida River, and one featuring actors titled, ‘Catching Fireflies by the Sumida River’ (T79). The Sumida River was also referenced in a print by Kiyonaga (T56). Other places referenced in the artwork included: Sahô River (T117), Koromogawa river at Tennōji-shita (T50), Ochanomizu (T23, T49), Ochiai (T89), Higashiyama and Yoneyama (T102), Sekiguchi (T132), Negishi Village in Toyko (T128), Mount Dōkan (T34), Chiyoda Castle (T12), Byodo-In Temple in Kyoto (T151), and Ichinose Bridge (T1, T172). The swarms of fireflies in the Valley of the Fireflies near Ishiyama and the lake of Ōmi were considered a natural marvel prior to 1703, but by 1903 people had noticed their populations declining [34] (pp. 143–144).

Image settings were primarily outdoors and featured water, most often streams or small rivers, and less frequently large rivers or lakes. Common plants featured in images included grasses, iris flowers (e.g., T14, T16, T29, T32, T44, T52, T54, T90), dwarf bamboo (T8), and willow trees (e.g., T9, T12, T112, T186). One 18th century garment (furisode kimono) at the National Museum of Japanese History with irises and fireflies was thought to be based on a 13th century poem by the shogun Minamoto no Sanetomo [44]. Hearn wrote, “*Fireflies frequent the neighbourhood of water, and like to circle above it; but some kinds are repelled by impure or stagnant water, and are only to be found in the vicinity of clear streams or lakes. The Genji-firefly shuns swamps, ditches, or foul canals; while the Heiké-firefly seems to be satisfied with any water. All fireflies seek by preference grassy banks shaded by trees; but they dislike certain trees and are attracted by others. They avoid pine trees, for instance; and they will not light upon rose-bushes. But upon willow trees—especially weeping willows—they gather in great swarms. Occasionally, on a summer night, you may see a drooping willow so covered and illuminated with fireflies that all its branches appear ‘to be budding fire’,*” [34] (pp. 151–152).

**Figure 4:**
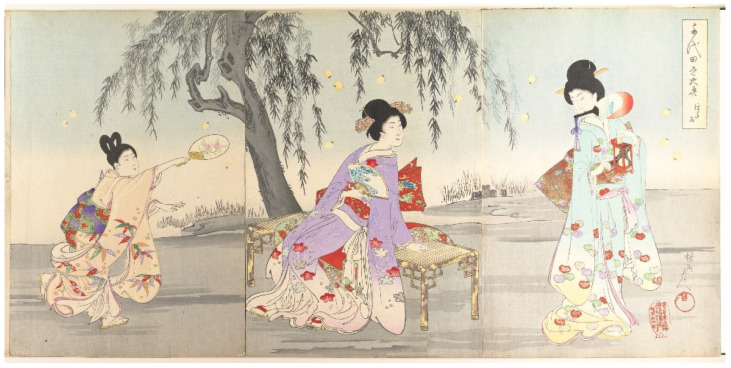
(T12). Chiyoda Castle (Album of Women). 1895. Yōshū (Hashimoto) Chikanobu (Japanese, 1838–1912). Triptych of woodblock prints; ink and color on paper. Gift of Mrs. W. Walton Butterworth, 1979. Accession Number: JP3547. The Metropolian Museum of Art, New York, NY, USA, www.metmuseum.org (accessed on from 4 January 2022). Open access image, CC0.

In many outdoor scenes there was evidence of human objects, such as benches (e.g., T51, T63, T73, T88, T158) some of which were quite ornate (T12, Figure 4), low-rise seating platforms (T70, T79, T85, T192), fences (T70, T89), stone walkways (T139), footbridges (e.g., T9, T18, T77, T90, T141), including a tall footbridge (T132), and decks or docks over water (T11, T14, T18, T87). Watercraft and dwellings were also common features, e.g., rafts (T20), sailboats (T69), other types of boats (e.g., T10, T60, T87, T133, T144-146, T154, T181), bridges (T67, T171) and buildings or houses (T14, T18, T48, T99, T109, T137).

Some images, especially larger triptych prints, had both an outdoor and indoor component, and included open air balconies or patios (e.g., T19, T56, T68-69, T96), gardens (e.g., T41, T109), or other open-air spaces adjoining houses (e.g., T9, T144-146, T182). These images often show people engaged in multiple types of activities, such as collecting fireflies, watching others collect, observing caged insects, or enjoying refreshments. It was less common to encounter images that were set solely indoors (T185), and occasionally the setting was unclear (T2), especially when only people and cages were shown (T26, T28, T45). In one Kunisada I print (T72), the only reference to fireflies was a picture of them hanging on a wall.

In modern-day Japan, watching adult fireflies is especially popular in early summer from May to July [30,48], and many prints referenced summer or a specific month in their titles, e.g., fifth month (T156), seventh month or July (T13, T96, T116), including a calendar print (T13) from July 1910 with an advertisement for ‘Deer and Stag’ pure silk from the Kawamata Silk Refining Company, Yokohama, Japan.

Several woodblock prints showed people collecting fireflies during a partial or full moon (e.g., T43, T46, T56, T68, T69, T102, T109, T132, T169, T171). Ambient light levels influence firefly behavior [49] although firefly abundance is similar during full and new moon phases [50,51]. However, artificial light pollution negatively impacts flashing activities and mating success of some firefly species and is considered a threat to firefly populations and conservation [52,53,54]. Hearn [34] (p. 152) indicated, “*During a bright moonlight night fireflies keep as much as possible in shadow […]. Lamplight, or any strong artificial light, drives them away; but small bright lights attract them. They can be lured, for example, by the sparkling of a small piece of lighted charcoal, or by the glow of a little Japanese pipe, kindled in the dark. But the lamping of a single lively firefly, confined in a bottle, or cup, of clear glass, is the best of all lures.*”

### 3.3. People Represented in Artwork

**Figure 5:**
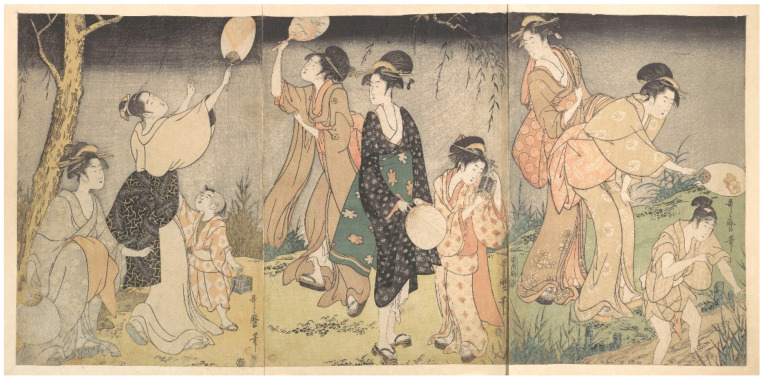
(T186). Catching fireflies (Hotaru gari). ca. 1796–97. Kitagawa Utamaro (Japanese, ca. 1754–1806). Triptych of woodblock prints; ink and color on paper. Rogers Fund, 1914. Accession Number: JP151. The Metropolian Museum of Art, New York, NY, USA, www.metmuseum.org (accessedon 4 January 2022). Open access image, CC0.

Japanese children frequently spend a great deal of time learning about, and observing or playing with insects, or *mushi*, and often hunt fireflies in the summer [34,48,55,56,57]. “*Girls follow the chase with paper fans; boys, with long light poles to the ends of which wisps of fresh bamboo-grass are tied.*” [34] (p. 150). This is not restricted to Japan, as Liu [58] notes, *“The fireflies are still one of the best evening entertainments the Chinese children have today. Mothers are generally requested by their children to save their empty egg-shells in which the youngsters house their catch and watch the flashing in the dark when they go to bed.*” Catching fireflies is also a common pastime for children in the United States [59,60,61,62,63]; Carter’s image ‘Fireflies’ showed two boys standing in water looking at fireflies trapped in a glass jar [64]. Some Japanese artwork only featured children (T39, T48, T195-196, T201), including Shuntei’s print with five girls, where the artist captured a sense of vigorous movement and excitement (T143). Most of the images with children showed one child collecting fireflies with one woman who was likely their parent (e.g., T16, T31, T43, T89, T138) or walking home after collecting (T132). There were also multiple women with one child (T42, T90) and multiple women with multiple children (e.g., T9, T11, T44, T109, T140-141), including ‘Catching Fireflies (Hotaru gari)’ by Utamaro (T186, Figure 5).

**Figure 6:**
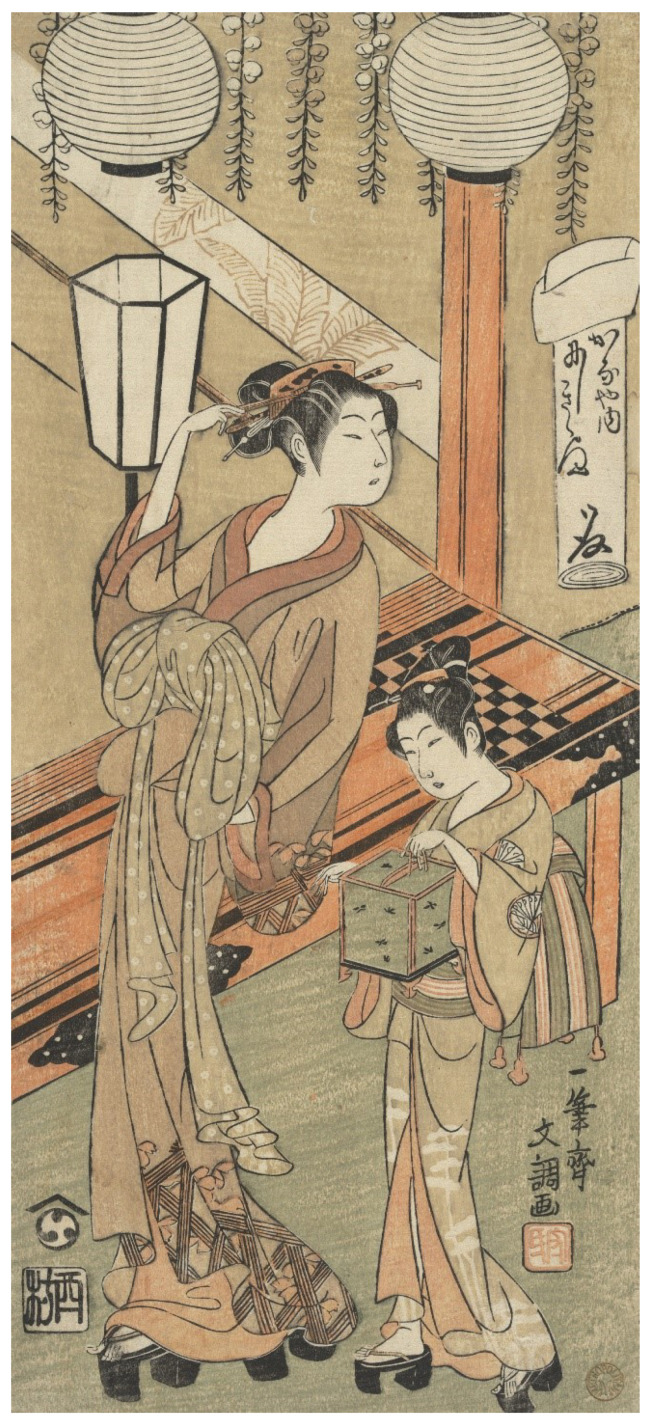
(T2). Courtesan and Attendant with a Cage of Fireflies. ca. 1770. Ippitsusai Bunchō (Japanese, active ca. 1765–1792). Woodblock print; ink and color on paper. Gift of Estate of Samuel Isham, 1914. Accession Number: JP907. The Metropolian Museum of Art, New York, NY, USA, www.metmuseum.org (accessed on 4 January 2022). Open access image, CC0.

Beautiful women (*bijin*), *geisha* (professional female entertainers), and courtesans were common subjects of *ukiyo-e* prints [35,37]. Solitary women were typically pictured catching or watching fireflies, and while it is unclear if some were considered *bijin* (e.g., T26, T32, T37, T41, T51-52, T98, T108, T135), other artworks were labeled as beauties (e.g., T22, T47, T58, T77, T80, T109, T120, T139, T142, T159), including several prints by Shoen (T122-123, T125). There were also prints of beauties with fireflies in the first collection of Modern Beauties (T118) and second series of Modern Beauties (T71, T119). Some pieces showed two women (e.g., T17, T29, T40, T158) and triptychs often featured one or two women in each panel, with some labeled as beauties; these images may also have shown women in different social classes, especially in scenes with larger numbers of women (T12, T20, T69, T73-74, T77, T92-94, T97). Women in a few prints were specifically referred to as *geisha* (T14, T106, T193) or courtesans (T2, Figure 6), and there were only a few more explicitly sexual images, including a woman showing her leg (Kiyomitsu I, T54), a see-through kimono (Yoshitoshi, T193), women with exposed nipples or bare breasts (Utamaro, T184-185, T187) and additional frontal nudity (Kiyomitsu, T53).

In Japan fireflies are a symbol of courtship [47], but relatively few images had a single man and woman (e.g., T63, T121, T180), and in Shōsō’s ‘Watching Fireflies on a Summer Night (T129) the man holds a knife behind his back. Couples were often featured in prints based on the famous Japanese story titled, ‘The Tale of Genji (*Genji monogatari*)’ (T36, T78, T97, T192), but not always (T67, T68, T75, T91, T101; Figure 3b). Chapter 25 in the story is called ‘Hotaru’ (fireflies), in which Genji released fireflies so that his brother Prince Hotaru can see his beloved Lady Tamakarura [65]. Other pieces with men focus on *kabuki* actors, which were extremely popular *ukiyo-e* prints [35]. Kunisada I created multiple pieces featuring actors (T79, T81-87), as well as Kiyomitsu I (T55), Kiyotsune (T57), Kunichika (T67), Kunihiro (T70), and Yoshitsuya (T194). One print (T150) was from the 1950s ‘Calendar of Kabuki Actors, with July featuring Lady Kasane,’ which showcased an *onnagata* (female impersonator); during that time period in *kabuki* theaters female roles were played by men [35,66]. It was uncommon to see images that only featured men that were not linked to the Genji story or actors (T34, T117).

**Figure 7:**
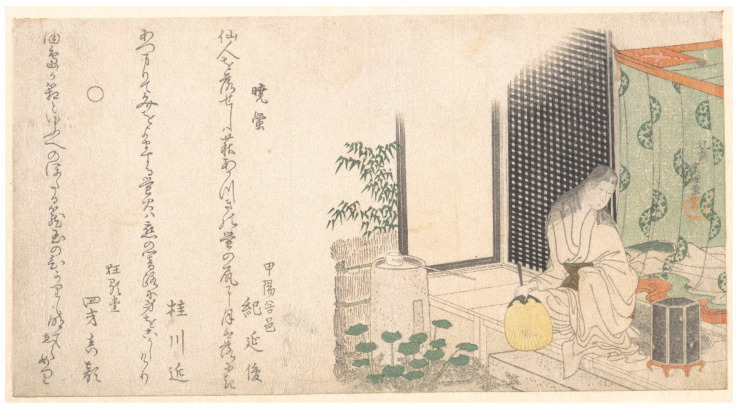
(T41). Cage of Fireflies at Dawn in Summer. ca. 1800. Katsushika Hokusai [Japanese, Tokyo (Edo) 1760–1849]. Woodblock print (surimono); ink and color on paper. The Howard Mansfield Collection, Purchase, Rogers Fund, 1936. Accession Number: JP2577. The Metropolian Museum of Art, New York, NY, USA, www.metmuseum.org (accessed on 4 January 2022). Open access image, CC0.

Images with larger groups of people showed social gatherings where the evening’s entertainment focused on collecting and observing fireflies. Most images had women and men, although sometimes only members of one sex were present, and occasionally children were also in attendance (e.g., T19-20, T56, T70, T88, T112, T128, T144-146). Lanterns, blankets, food, beverages, pipes, musical instruments, and pets were often pictured, emphasizing the recreational aspect. Artists rarely pictured individuals wading in the water while collecting (T18, T186). However, small groups in boats (T10, T60, T133) and larger boating parties were common (T107, T154, T181). ‘Firefly Viewing Party’ shows a boating party near a bridge where other people were also catching fireflies, and the museum commentary reads, “*Hotarugari (firefly viewing) is a popular summer pastime in Japan. These small insects produce flashes of light-which can be seen at night-during the hot months when they breed. Since they live near water, firefly viewing had an added attraction: the cool night breezes off the water brought relief from the heat. The people depicted in this print have hired a boat to take them out on the water. Those in the bow reach towards the fireflies with their fans, attempting to sweep them closer. Those in the center of the boat are drinking and chatting convivially. An attendant blows on a portable stove, attempting to keep a small fire alive so that he can prepare a snack for the group. Twenty-nine haiku poems on the theme of summer are printed in the upper portion of the print.*” (Minneapolis Museum of Art, T171). This artwork is considered a *surimono*, or genre of non-commercial woodblock prints that pair illustration with text, which were often used as private announcements for special events [67]. Other *surimono* featuring fireflies included: T41 (Figure 7), T43-44, T138, and T171.

A few images were humorous and involved physical comedy. Hirokage’s ‘Catching Fireflies at Mount Dōkan’ (T34) showed fireflies flying around four men drinking alcoholic beverages. One *sumizuri-e* print (T180) pictured a standing woman reaching for a firefly and an upended bench with a man tumbling unceremoniously to the ground. ‘Thirty-six Amusing Views of Famous Places in Tokyo: Negishi Village’ by Ikkei (T128) depicted a man falling into a stream and fireflies escaping from his airborne cage.

Artwork showing fireflies being used for practical purposes was rare. The design on a kozuka (T147) showed a man reading by the light of a suspended bag of fireflies, and Hearn [68] (p. 459) mentioned, “*[…] story of that Chinese student who, being too poor to pay for a lamp, imprisoned many fireflies in a paper lantern, and thus was able to obtain light enough to study after dark, and to become eventually a great scholar.*” The museum description of a Chinese painted folding fan featuring multiple men reads, “*A firefly lamp hangs from a branch to light the scrolls upon the table.*” [69].

### 3.4. Tools to Collect and Cage Fireflies

Folding sensu fans and flat uchiwa fans were used to collect fireflies; some were plain but many had ornate designs, including what looks like an actor’s face (T77). Uchiwa fans on long poles (T9-10, T44, T88, T109, T112, T128, T140-141, T143-146), or long bamboo poles with leaves at the top are also commonly pictured (T9, T11, T25, T60-61, T110, T128, T138, T140, T154, T157), although nets of any kind were rare (T29) and may not always be linked to fireflies (T152). People, especially children, also used their hands to capture the insects (e.g., T37, T39, T90, T156). One print (Gesso, T25) showed collection tools and fireflies in a cage in the absence of people. The museum description of the Shinsui print ‘Firefly’ says, “*A young woman is about to bat a firefly with her round fan. She will then place the paralyzed firefly into a cage and collect more to release them all at once later.*” (Minneapolis Institute of Art, https://collections.artsmia.org/art/62344/firefly-ito-shinsui (accessed on 4 January 2022), a different print of T119).

**Figure 8:**
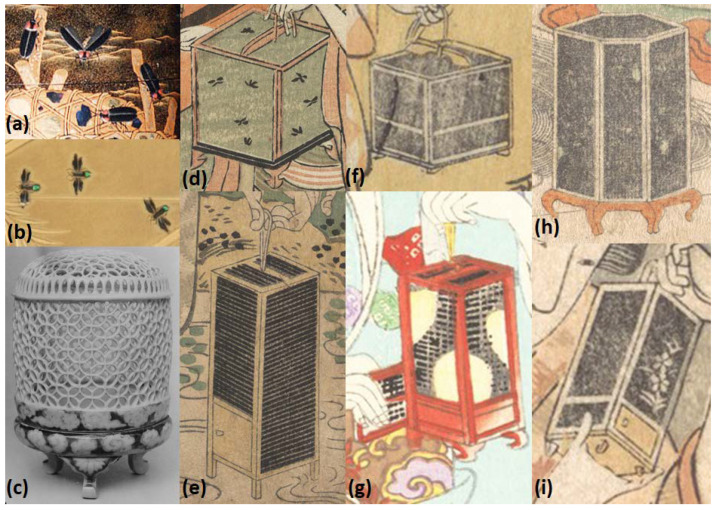
Examples of fireflies and firefly cages: (**a**) detail from Figure 1, (**b**) detail from T163, (**c**) T65 (cropped), (**d**) detail from Figure 6 (T2), (**e**) detail from Figure 3a (T16), (**f**) detail from Figure 5 (T186), (**g**) detail from Figure 4 (T12), (**h**) detail from Figure 7 (T41), (**i**) detail from Figure 5 (T186). The Metropolian Museum of Art, New York, NY, USA, www.metmuseum.org (accessed on 4 January 2022). Open access images, CC0.

**Figure 9:**
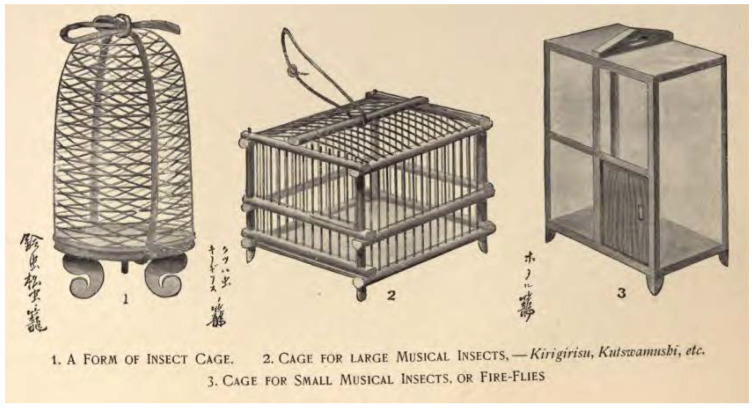
Insect cages. Hearn, L. *Exotics and Retrospectives*. Little, Brown, and Co.: Boston, MA, USA, 1898, pp. 50–51. [70].

A wide variety of firefly cages were represented, both in shape, size, and style (Figure 8), and one lacquer box was designed so that it looked like a cage full of fireflies, complete with faux mesh and glowing insects (T176). Cage shapes ranged from cubes, rectangles, tall hexagonal cages, and various types of cylinders, including domes. Figure 8c shows a cylindrical firefly cage made of porcelain with an interlocking pattern of circles (T165). Hearn [34] (p. 148) noted, “*The cheapest kind of cage, containing only three or four fireflies, is scarcely more than two inches square; but the costly cages—veritable marvels of bamboo work, beautifully decorated—are as large as cages for song-birds. Firefly cages of charming or fantastic shapes—model houses, junks, temple-lanterns, etc.—can be bought at prices ranging from thirty sen up to one dollar.*” Cages occurred in many colors, e.g., black, brown, red, yellow, white. Most cages had legs on the bottom and a door to add and release fireflies, and people carried them by a cord attached to the top. The cage in Kuniyoshi’s ‘Catching Fireflies (T93-94) was incredibly large and ornate, with multiple designs and red tassels. In contrast, an image from the 1950s (T48) showed boys with a cage fashioned from a gourd, although this may be a non-specific insect cage. Firefly cages were typically shown with a tight-weave mesh that occasionally had a floral design (e.g., T89, T95, T113-114). The fine mesh appeared to distinguish firefly cages from other insect cages, such as those for ‘singing’ insects such as orthopterans, perhaps because the latter can typically chew through fabric mesh (Figure 9).

### 3.5. Collecting Other Insects

Many Japanese woodblock prints and objects focused on insects other than fireflies [44], particularly Orthoptera. Some featured insects (T66), images of cages or actual cages (T111, T153, T173, T178-179, T204), people with cages (T33, T64) or people collecting, such as ‘Famous Places in the Eastern Capital—Listen to Singing of Insects at Dokanyama Hill’ (Hiroshige, T38) and ‘Ladies Imitating a Courtly Insect Hunt’ (Eisen, T21).

**Figure 10:**
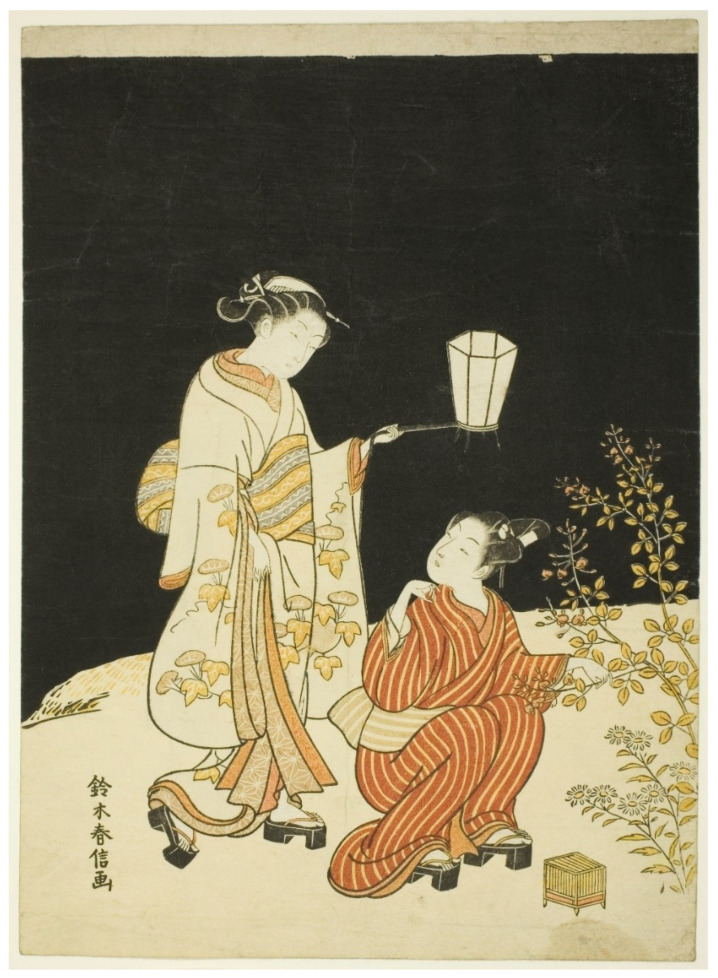
(T30). Searching for Fireflies. ca. 1768. Suzuki Harunobu (Japanese, 1725–1770). Color woodblock print, chūban. Clarence Buckingham Collection. Reference Number: 1952.327. The Art Institute of Chicago, IL USA, https://www.artic.edu/artworks/77297/searching-for-fireflies (accessed on 4 January 2022). Open access image, CC0.

Based on the morphology of the insects in the images, the cage style, and the surrounding vegetation, artwork labeled as relating to fireflies might actually involve Odonata or Orthoptera. The insects in ‘Mother and Children Enjoying Fireflies’ (Utamaro, T187) appear to be damselflies or dragonflies, and the child is swatting at them with a thin rod rather than the tools typically used to collect fireflies. Misidentifications were probably due to misinterpretations by people other than the artists, possibly when English titles were added to the artwork. The museum description for a sake dish (T46) reads, “*[…] seven women among autumn plants under a partial moon with three holding an insect cage each trying to catch insects, most probably fireflies, while a group of three men, including two samurai, walk towards the women […].*” However, the insect pictured in the upper left corner looks like a tree cricket, and there is a distinctive shrub that is pictured in other images where it seems likely that people are hunting Orthoptera instead of fireflies (T21, T30, Figure 10). In some cases, it’s difficult to discern if insects or cages relate to Orthoptera or fireflies, e.g., Utamaro’s print ‘Picture of the Upper Class’ (T188), Kuniyoshi’s ‘Woman with Fan and Insect Cage (T98), and Zeshin’s ‘Fan and Insect Cage’ (T205). Artwork with a likely or definite connection to insects other than fireflies is distinguished in Table A1 by letter codes after the title (see Table A1’s footnote 1 for more details).

### 3.6. Selling Fireflies

People collected fireflies for personal entertainment, but fireflies were also collected and sold en masse as a commodity [27]. The majority of fireflies for sale in the Japanese modern monarchical period (1868–1945) were field collected and were primarily the larger Genji-boturu (*Luciola cruciata*) [34,47]. Hearn [34] (pp. 144–146) described in detail how professional firefly-catchers obtained hundreds to thousands of fireflies each night near the Lake of Ōmi to supply large cities such as Kyōto and Ōsaka: “*Immediately after sunset the firefly-hunter goes forth with a long bamboo pole upon his shoulder, and a long bag of brown mosquito-netting wound, like a girdle, about his waist. When he reaches a wooded place frequented by fireflies,—usually some spot where willows are planted, on the bank of a river or lake,—he halts and watches the trees. As soon as these begin to twinkle satisfactorily, he gets his net ready, approaches the most luminous tree, and with his pole strikes the branches. The fireflies […] drop helplessly to the ground […] the catcher, picking them up with astonishing quickness, using both hands at once, deftly tosses them into his mouth—because he cannot lose the time required to put them, one by one, into the bag. Only when his mouth can hold no more, does he drop the fireflies, unharmed, into the netting. Thus the firefly-catcher works until about two o’clock in the morning,—the old Japanese hour of ghosts,—at which time the insects begin to leave the trees and seek the dewy soil. There they are said to bury their tails, so as to remain viewless. But now the hunter changes his tactics. Taking a bamboo broom he brushes the surface of the turf, lightly and quickly. Whenever touched or alarmed by the broom, the fireflies display their lanterns, and are immediately nipped and bagged. A little before dawn, the hunters return to town.*” Fireflies were sorted by the intensity of the light they produced, then several hundred stored in gauze-covered boxes or cages along with moistened grass or turf sprinkled with fresh water [34,71].

Mass-collecting fireflies was not restricted to Japan. In the United States in the 1950s–1980s, children and young biologists collected tens of millions of fireflies for professors, universities and chemical companies, with children recruited to the Sigma Firefly Scientists Club receiving a penny apiece for their bounty [72,73,74].

Laurent [55] indicated that around 1685 in Kyoto insect sellers would carry their wares, specifically singing crickets, in baskets suspended from poles worn across their shoulders. This is akin to Zeshin’s print ‘Insects Seller’ featuring an orthopteran (T204), where two large cages, from which small cages are suspended, hang from a padded pole. Pushcarts that sold insects and cages appeared around 1820 followed by “*mushiya, or shops that sold singing insects, fireflies, and jewel beetles as well as cages and trapping devices,*” in the Meiji period (1868–1912) [55].

Prints or photos of insect sellers included a variety of cages, with and without insects, displayed in what appear to be portable stalls with poles or straps (T3, T57), folding stalls (T28, T76, T189) and indoor or outdoor stands (T114, T137, T190). Several of the stalls or stands had a checkerboard design (T28, T76, T114, T131, T190). Images showed children (T190), women and children (T28, T114, T137), actors as vendors (T57, T76), or lone vendors (T3). Shōtei’s ‘Insect Seller’ (T131) is particularly poignant, with a child in silhouette holding out her firefly cage to a vendor sitting in front of outdoor stall as bats fly overhead.

Hearn [75] (pp. 86–87) described an insect seller at the Market of the Dead, “*Hotaru-ni-kirigisu! […] A little booth shaped like a sentry-box, all made of laths, covered with a red-and-white chess pattern of paper; […] there are also beautiful little cages full of fireflies,—cages covered with brown mosquito-netting, upon each of which some simple but very pretty design in bright colours has been dashed by a Japanese brush. One cricket and cage, two cents. Fifteen fireflies and cage, five cents.*” Hoshina [47] reported that based on information in newspapers, the price of a firefly was extremely inexpensive: 0.1 Japanese sen in 1886, and 5 sen in 1917, with 100 sen equal to 1 yen. Hearn [34] (pp. 147–148) wrote, “*the wholesale price of living fireflies ranges from three sen per hundred up to thirteen sen per hundred, according to season and quality. Retail dealers sell them in cages; and in Tokyo the price of a cage of fireflies ranges from three sen up to several dollars.*” However, in 2017, the price of a firefly was much higher, perhaps due to their declining populations, and was listed as 400 yen [47].

Fireflies were sold to individuals, restaurants, hotels, and wholesale and retail insect-merchants [27,34,47]. Fireflies were released at events honoring military victories and royalty and were given out by retailers as free gifts to entice customers [47,59]. “*In the famous Dōtombori of Ōsaka, there is a house where myriads of fireflies are kept in a large space enclosed by mosquito-netting; and customers of this house are permitted to enter the enclosure and capture a certain number of fireflies to take home with them.*” [34] (p. 147).

People often purchased fireflies at pet shops or summer festivals that were kept in cages until the insect died [47]. Fireflies that died in insect-shops still had value and were used in the formulation of drugs and ointments or firefly grease (*Hotaru-no-abura*) used by woodworkers [34]. Some individuals purchased large quantities of fireflies to release at evening parties or events so that guests could enjoy their sparkling lights [34]. However, one woman wrote, “*May 18th 1889. I went to a night fair […] there was one stall full of winged lights, tiny stars of green fire clustering all over it. I bought about a hundred Princess Splendours in a black horsehair cage, and brought them home with me. […] But the keeper of the strange stall at the fair (and I could hardly see it for the darkness) had captured scores of the winged lights, and sold them by ones and twos in a dainty cage two inches long, with a green leaf for provisions, for two rin, a sum so small that we have no equivalent for it. I stood for a minute before the firefly stall, and then told the interpreter to say that I must have all the fireflies in all the cages. […] I carried them all home in the horsehair box; and when everybody had gone to bed, I crept out into the balmy darkness of my garden, opened the box, and set all the lovely creatures free.*” [76] (pp. 38–41).

## 4. Discussion

This study summarizes the diversity in representations of fireflies and insect–human interactions by a multitude of Japanese artists. Watching and catching fireflies was and continues to be a recreational pastime in many parts of the world, although selling fireflies is rare [47,56]. In Japan, selling insects declined in the 1930s and *mushiya* were rare by the end of World War II [55], although there is still interest in insects as a commodity [77,78]. Insects such as fireflies remain an important part of Japanese culture [31,47,56], and are protected by legal and social policies [27]. Firefly ecotourism (e.g., celebrations, festivals, tours of firefly sanctuaries, firefly ‘villages’), has emerged in multiple countries, which can serve as a potential tool to educate the public about firefly conservation and threats to their populations, including habitat degradation, water and light pollution, and insecticides [10,23,29,79,80,81,82,83,84]. Ensuring that these revenue-generating activities do not negatively impact fireflies or their habitat is an important component of sustainable firefly ecotourism [10,82,83,84,85]. The concept and definition of environmental art, or eco-art, has evolved throughout time, but in general eco-art education is a multidisciplinary approach that integrates art, science, and education with a focus on the environment, including biodiversity, conservation, sustainability, and restoration [86,87,88]. Using art to highlight the historical and cultural significance of fireflies may also help with conservation efforts.

## Data Availability

Not applicable.

## Appendix A

**Table A1:** List of artwork (accessed multiple times from 4 January 2022 to 22 August 2022).

ID #	Artist	Title ^1^	Type ^2^	Date	Site ^3^
1	Beato (Beath), Felice (1834–?)	Ichinose Bridge of Nakashimagawa River (Tea House […]) (Z) http://oldphoto.lb.nagasaki-u.ac.jp/global/search/jp_detail.php?id=1289	PH	1864	NUL
2	Bunchō, Ippitsusai (1727–1796)	Courtesan and Attendant with a Cage of Fireflies (S) https://www.metmuseum.org/art/collection/search/51997	W	ca.1770	MET
3	Bunchō, Ippitsusai (1727–1796)	Insect Vendor (20th c. repro) (Z, FN) https://honolulumuseum.org/collections/9312/	W	18th c.	HM
4	Bunrin, Shiokawa (1808–1877)	River Landscape with Fireflies (1) (Z, G) https://art.nelson-atkins.org/objects/15298/river-landscape-with-fireflies	PA, R	1874	NAM
5	Bunrin, Shiokawa (1808–1877)	River Landscape with Fireflies (2) (Z, G) https://art.nelson-atkins.org/objects/23679/river-landscape-with-fireflies	PA, R	1874	NAM
6	Bunrin, Shiokawa (1808–1877)	Fireflies by a Twilight Stream (L, G) https://emuseum.mfah.org/objects/71741/fireflies-by-a-twilight-stream	PA	ca.1875	MFAH
7	Bunrin, Shiokawa (1808–1877)	Fireflies at River’s Edge (L, G) https://collections.lacma.org/node/2158943	PA	19th c.	LACMA
8	Bunrin, Shiokawa (1808–1877)	Fireflies Over River (A, G) https://www.britishmuseum.org/collection/object/A_1980-0728-0-4	PA	19th c.	BRM
9	Chikanobu, Yôshû (1838–1912)	Fireflies at a Country House (Summerhouse) (S, G) http://www.jaodb.com/db/ItemDetail.asp?item=34139	W, T	ca.1880	UKIYO
10	Chikanobu, Yôshû (1838–1912)	Chasing Fireflies (S, G) https://ukiyo-e.org/image/artelino/8338g1	W	1893	UKIYO
11	Chikanobu, Yôshû (1838–1912)	Summer - Women and Children Catching Fireflies (S, G) https://ukiyo-e.org/image/jaodb/Chikanobu_Yoshu-Songs_of_the_Four_Seasons-Summer_Women_and_children_catching_fireflies-00041561-080908-F12	W, T	1894	UKIYO
12	Chikanobu, Yôshû (1838–1912)	Chiyoda Castle (Album of Women) (S, G) https://www.metmuseum.org/art/collection/search/55848	W, T	1895	MET
13	Chikanobu, Yôshû (1838–1912)	Calendar Print for July 1910 (D) https://collections.mfa.org/objects/155613	W	1909	MFAB
14	Chikanobu, Yôshû (1838–1912)	Geisha Sakyo of Hikota-ro and Another Geisha of Nakanocho (S, G) https://ukiyo-e.org/image/metro/0793-K002-009	W	1838–1912	UKIYO
15	Chikanora (active 1900–1920)	Girl Holding an Insect Cage (Z) https://asia.si.edu/object/S2003.8.82/	W	1900–1920	SNMAA
16	Chōki, Eishōsai (active ca.1790s–early 1800s)	Woman and Child Catching Fireflies (S, G) https://www.metmuseum.org/art/collection/search/44993	W	ca.1793	MET
17	Eisen, Keisai (1790–1848)	Two Women Catching Fireflies (Z) https://collections.mfa.org/objects/26650	PA	ca.1818–1844	MFAB
18	Eisen, Keisai (1790–1848)	A Modern Firefly Hunt (S) https://ukiyo-e.org/image/famsf/7224328201820058	W, T	1820	UKIYO
19	Eishi, Chôbunsai (1756–1829)	Fireflies (L) https://www.loc.gov/pictures/collection/jpd/item/2008661240/	W, T-c, r	1789 or 1790	LOC
20	Eishi, Chôbunsai (1756–1829)	Women Catching Fireflies (L) https://collections.mfa.org/objects/502283	W, T	ca.1796–1797	MFAB
21	Eishi, Chôbunsai (1756–1829)	Ladies Imitating a Courtly Insect Hunt (N) https://collections.mfa.org/objects/489223	W, T	1756–1829	MFAB
22	Eizan, Kikukawa (Kikugawa) (1787–1867)	Bijin and Firefly Cage (20th c. repro) (Z) https://ukiyo-e.org/image/artelino/10440g1	W	1600–1868	UKIYO
23	Farsari, Adolfo (1841–1891)	Ochanomizu and Hijiri-bashi Bridge (Ochiyanomizu, Tokyo) (Z) http://oldphoto.lb.nagasaki-u.ac.jp/global/search/jp_detail.php?id=4196	PH	1892–1897	NUL
24	Gekkō, Ogata (1859–1920)	Fireflies on the River (S, G) https://asia.si.edu/object/S2003.8.1780/	W	ca.1890–1910	SNMAA
25	Gesso, Yoshimoto (1831–1936)	Catching Firefly’s (D) https://asia.si.edu/object/S2003.8.3644/	W	1831–1936	SNMAA
26	Goyō, Hashiguchi (1881–1921)	Woman with Firefly Cage (L) https://honolulumuseum.org/collections/3842/	W	1920	HM
27	Hamano School	Fireflies and Grasses (kozuka) (A) https://collections.mfa.org/objects/12311	O	ca. mid-19th c.	MFAB
28	Harumasa, Koikawa (Banki), active (1800–1820)	Young Mother […] Buying a Firefly Box (Z, FN) https://www.britishmuseum.org/collection/object/A_1906-1220-0-250	W	1800–1820	BRM
29	Harunobu, Suzuki (1724–1770)	Catching Fireflies (L) https://www.artic.edu/artworks/20977/catching-fireflies	W	ca.1767	AIC
30	Harunobu, Suzuki (1724–1770)	Searching for Fireflies (Z, NP) https://www.artic.edu/artworks/77297/searching-for-fireflies	W	ca.1768	AIC
31	Harunobu, Suzuki (1724–1770)	Catching Fireflies (L, G) https://honolulumuseum.org/collections/2213/	W	ca.1765–1770	HM
32	Harunobu, Suzuki (1724–1770)	Young Woman Chasing Fireflies with a Fan (L) https://collections.mfa.org/objects/178047	W	ca.1768–1769	MFAB
33	Harunobu, Suzuki (1724–1770)	Beauty with Attendant Carrying an Insect Cage (NP) https://collections.artsmia.org/art/63510	W	1760s	MIA
34	Hirokage, Utagawa (active ca.1850s–1860s)	Catching Fireflies at Mount Dōkan (L, G) https://www.loc.gov/pictures/collection/jpd/item/2008660927/	W	1859	LOC
35	Hiromitsu, Nakazawa (1874–1964)	Firefly and Floral Rondel from Chûgaku Sekai (S) https://collections.mfa.org/objects/566112	L	1905	MFAB
36	Hiromitsu, Nakazawa (1874–1964)	Firefly - The Tale of Genji (S, G) https://ukiyo-e.org/image/artelino/32064g1	W	1912	UKIYO
37	Hiroshige, Utagawa (1797–1858)	Catching Fireflies (S) https://www.artic.edu/artworks/47700/catching-fireflies	W	1797–1858	AIC
38	Hiroshige, Utagawa (1797–1858)	Listen to Singing of Insects at Dokanyama Hill: Famous Places […] (Z, NP) https://www.edohakuarchives.jp/detail-174.html	W	1839-–1842	TM
39	Hitoshi, Kiyohara (1896–1956)	Catching Fireflies (D) https://collections.lacma.org/node/190563	W	mid-20th c.	LACMA
40	Hokuba, Teisai (1771–1844)	Women Chasing Fireflies (S) https://emuseum.mfah.org/objects/9000	PA	1771–1844	MFAH
41	Hokusai, Katsushika (1760–1849)	Cage of Fireflies at Dawn in Summer (D) https://www.metmuseum.org/art/collection/search/54193	W	ca.1800	MET
42	Hokusai, Katsushika (1760–1849)	Women Catching Fireflies (S) https://collections.mfa.org/objects/209821	W	1760–1849	MFAB
43	Hokusai, Katsushika (1760–1849)	Cloth Fulling (Z, NP) https://www.fujiarts.com/cgi-bin/item.pl?item=956391#top	W	1760–1849	FUJI
44	Hokusai, Katsushika (1760–1849)	Catching Fireflies (S) https://www.sothebys.com/en/buy/auction/2022/fine-japanese-prints/katsushika-hokusai-1760-1849-catching-fireflies	W	1798	SOT
45	Hokushi (mid 19th c.)	Two Women, One Carrying Insect Cage and Fan […] (Z) https://www.britishmuseum.org/collection/object/A_1881-1210-0-1786	PA	19th c.	BRM
46	Kajikawa	Sake Dish (NP) https://collections.vam.ac.uk/item/O434852/sake-dish-kajikawa/	O	ca.1775–1850	VAM
47	Keishū, Takeuchi (1861–1942)	A Beauty Hunting Fireflies (S) https://honolulumuseum.org/collections/7945/	W	1897	HM
48	Keishū, Takeuchi (1861–1942)	Looking for Insects (NP) https://ukiyo-e.org/image/jaodb/Keishu_Takeuchi-No_Series-Looking_for_Insects-00039949-061216-F12	L	1910–1920s	UKIYO
49	Kiyochika, Kobayashi (1847–1915)	Fireflies at Ochanomizu (D) https://honolulumuseum.org/collections/1626/	W	1879	HM
50	Kiyochika, Kobayashi (1847–1915)	Koromogawa River at Tennōji-shita (D) https://www.si.edu/object/koromogawa-river-tennoji-shita:fsg_S2003.8.1176	W	1880	SNMAA
51	Kiyohiro, Torii (active 1737–1776)	Young Woman Catching Fireflies on a Fan (A) https://collections.mfa.org/objects/233095	W	ca.1745–1755	MFAB
52	Kiyomitsu I, Torii (1735–1785)	Young Woman Chasing Fireflies (L) https://collections.mfa.org/objects/176427	W	1735–1785	MFAB
53	Kiyomitsu I, Torii (1735–1785)	Beauty Catching Fireflies (20th c. repro) (S, G) https://ukiyo-e.org/image/artelino/44589g1	W	ca.1750	UKIYO
54	Kiyomitsu I, Torii (1735–1785)	Chasing Fireflies (L) https://www.artic.edu/artworks/23267/chasing-fireflies	W	ca.1761	AIC
55	Kiyomitsu I, Torii (1735–1785)	Two Actors Catching Fireflies (L) https://www.artic.edu/artworks/19936/two-actors-catching-fireflies	W	ca.1765–1770	AIC
56	Kiyonaga, Torii (1752–1815)	A Party Viewing the Moon across the Sumida River (S) https://collections.mfa.org/objects/497734	W, T	ca.1787	MFAB
57	Kiyotsune, Torii (active 1757–1779)	Actor Nakamura Tomijûrô I as an Insect Vendor (Z, NF) https://collections.mfa.org/objects/211824	W	ca.1774	MFAB
58	Kodou, Yamanaka (1869–1945)	Firefly (L, G) https://ukiyo-e.org/image/jaodb/Kodo_Yamanaka-No_Series-Firefly-00043093-110814-F06	W	1913	UKIYO
59	Kōgyō, Tsukioka (1869–1927)	Fireflies (A, G) https://asia.si.edu/object/S2003.8.2937/	W	1940s	SNMAA
60	Koho, Shoda (1870–1946)	Catching Fireflies (L, G) https://ukiyo-e.org/image/artelino/47049g1	W	ca.1930s	UKIYO
61	Kōryū, Koma (?–1796)	Case (Inrō) with Design of People Catching Fireflies (S, G) https://www.metmuseum.org/art/collection/search/45575	O	19th c.	MET
62	Koryûsai, Isoda (1735–1790)	Couple Watching Fireflies (L) https://honolulumuseum.org/collections/2631/	W	ca.1770	HM
63	Koryûsai, Isoda (1735–1790)	Young Couple Watching Fireflies (Fashionable Twelve Months series) (L) https://collections.mfa.org/objects/176244	W	ca.1770–1772	MFAB
64	Koryûsai, Isoda (1735–1790)	Young Woman Hanging a Mosquito Net, with Insect Cage and Cat (N) https://collections.mfa.org/objects/176357	W	1735–1790	MFAB
65	Koson, Ohara (1878–1945)	Fireflies (A, G) https://asia.si.edu/object/S2003.8.2056/	W	ca.1927	SNMAA
66	Kōzan I, Makuzu (1842–1916)	Freshwater Jar (Mizusashi) with Procession of Grasshoppers (X) https://www.metmuseum.org/art/collection/search/53613	O	ca. 1870–1880s	MET
67	Kunichika, Toyohara (1835–1900)	Modern Genji – Firefly Viewing (center) (S, G) https://www.metmuseum.org/art/collection/search/58253	W, T-c	1861	MET
68	Kunichika, Toyohara (1835–1900)	Chapter 25: Hotaru (D) https://honolulumuseum.org/collections/8571/	W	ca.1884	HM
69	Kunichika, Toyohara (1835–1900)	Chasing Fireflies (S, G) https://ukiyo-e.org/image/jaodb/Hiroshige_2_and_Kunichika-No_Series-Chasing_Fireflies-00035737-040111-F06	W, T	1835–1900	UKIYO
70	Kunihiro, Utagawa (active ca.1815–1843)	The Firefly Party (L) https://ukiyo-e.org/image/jaodb/Kunihiro_Utagawa_Ganjosai-No_Series-The_Firefly_Party-00041832-090118-F12	W, P	1823	UKIYO
71	Kunisada I, Utagawa (Toyokuni III) (1786–1864)	Catching Fireflies at Sekiya (Modern Beauties […] series) (Z) https://collections.mfa.org/objects/477665	W	ca.1822–1825	MFAB
72	Kunisada I, Utagawa (Toyokuni III) (1786–1864)	Rice Stalks and Fireflies (Collection of Fashionable Pairings series) (L) https://collections.mfa.org/objects/207581	W	1831	MFAB
73	Kunisada I, Utagawa (Toyokuni III) (1786–1864)	Catching Fireflies (left) (L, G) https://collections.mfa.org/objects/480946	W, T-l	1843–1847	MFAB
74	Kunisada I, Utagawa (Toyokuni III) (1786–1864)	Catching Fireflies (right) (L, G) https://collections.mfa.org/objects/480874	W, T-r	1843–1847	MFAB
75	Kunisada I, Utagawa (Toyokuni III) (1786–1864)	Fireflies in Darkness (L, G) https://collections.mfa.org/objects/477526	W, T	1847–1852	MFAB
76	Kunisada I, Utagawa (Toyokuni III) (1786–1864)	Insect Seller (Selected Six Sellers in the Summer Night series) (D, FN) https://www.edohakuarchives.jp/detail-320.html	W	1847–1852	TM
77	Kunisada I, Utagawa (Toyokuni III) (1786–1864)	Beauties Viewing Fireflies (L, G) https://ukiyo-e.org/image/harashobo/16704_3	W, T	ca.1848	UKIYO
78	Kunisada I, Utagawa (Toyokuni III) (1786–1864)	Ch. 25 Hotaru (The Color Print Contest of a Modern Genji series) (L, G) https://collections.mfa.org/objects/179450	W	1852	MFAB
79	Kunisada I, Utagawa (Toyokuni III) (1786–1864)	Catching Fireflies by the Sumida River: Actors […] (L) https://collections.mfa.org/objects/510125	W, T	1853	MFAB
80	Kunisada I, Utagawa (Toyokuni III) (1786–1864)	Firefly Hunting – Kabuki (L, G) https://ukiyo-e.org/image/artelino/34778g1	W	1855	UKIYO
81	Kunisada I, Utagawa (Toyokuni III) (1786–1864)	Catching fireflies resembling a collection of shining pearls (left) (L, G) https://ja.ukiyo-e.org/image/mak/15063-3	W, T-l	1855	UKIYO
82	Kunisada I, Utagawa (Toyokuni III) (1786–1864)	Catching fireflies resembling a collection of shining pearls (center) (L, G) https://ja.ukiyo-e.org/image/mak/15063-2	W, T-c	1855	UKIYO
83	Kunisada I, Utagawa (Toyokuni III) (1786–1864)	Catching fireflies resembling a collection of shining pearls (right) (L, G) https://ja.ukiyo-e.org/image/mak/15063-1	W, T-r	1855	UKIYO
84	Kunisada I, Utagawa (Toyokuni III) (1786–1864)	Imaginary Scene of Actors Catching Fireflies: Jewels Shining […] (L, G) https://collections.mfa.org/objects/510096	W, T	1855	MFAB
85	Kunisada I, Utagawa (Toyokuni III) (1786–1864)	Viewing Fireflies in the Cool of the Evening: Actors […] (L, G) https://collections.mfa.org/objects/471740	W, T-r	1859	MFAB
86	Kunisada I, Utagawa (Toyokuni III) (1786–1864)	Fashionable Firefly-Hunting: Actors […] (A, L, G) https://collections.mfa.org/objects/476126	W, TE	1860	MFAB
87	Kunisada I, Utagawa (Toyokuni III) (1786–1864)	Actors Onoe Kikujirô II as Akizuki’s Daughter […] (S, G) https://collections.mfa.org/objects/477698	W, D	1855	MFAB
88	Kunisada I, Utagawa (Toyokuni III) (1786–1864)	Catching Fireflies by the Uji River in Yamashiro Province (S, D, G) https://collections.mfa.org/objects/497844	W, T	1861	MFAB
89	Kunisada I, Utagawa (Toyokuni III) (1786–1864)	Fireflies at Ochiai (The Pride of Edo: Thirty-six Scenes series) (S, D, G) https://collections.mfa.org/objects/322515	W	1864	MFAB
90	Kunisada I, Utagawa (Toyokuni III) (1786–1864)	Yusuzumi Sawabe No Hotaru (L, G) https://www.britishmuseum.org/collection/object/A_1907-0531-0-215-1-3	W, T	1786–1864	BRM
91	Kunisada II, Utagawa (1823–1880)	CH25 – Fireflies (L, G) http://www.jaodb.com/db/ItemDetail.asp?item=29944	W	1857	UKIYO
92	Kuniyoshi, Utagawa (1797–1861)	Catching Fireflies in the Cool of the Evening ([…] Seasons series) (S) https://collections.mfa.org/objects/500174	W, T	1843–1847	MFAB
93	Kuniyoshi, Utagawa (1797–1861)	Catching Fireflies (possibly left sheet to #94) (D) https://ukiyo-e.org/image/artelino/34443g1	W, D-l?	1849–1853	UKIYO
94	Kuniyoshi, Utagawa (1797–1861)	Catching Fireflies in the Cool of the Evening (D) https://collections.mfa.org/objects/190492	W, D-r?	1847–1852	MFAB
95	Kuniyoshi, Utagawa (1797–1861)	Firefly Catching from the Three Hunts in This Country (L, G) https://www.edohakuarchives.jp/detail-1659.html	W	1847–1848	TM
96	Kuniyoshi, Utagawa (1797–1861)	The Seventh Month (Fumizuki) (L, G) https://collections.mfa.org/objects/494208	W, T	1847–1852	MFAB
97	Kuniyoshi, Utagawa (1797–1861)	Firefly (Scenes from The Tale of Genji series) (Z) https://emuseum.mfah.org/objects/86605	W	1797–1861	MFAH
98	Kuniyoshi, Utagawa (1797–1861)	Woman with Fan and Insect Cage (Z, NP) https://collections.artsmia.org/art/80938/woman-with-fan-and-insect-cage-utagawa-kuniyoshi	W	1844–1846	MIA
99	Kyosen, Kawasaki (1877–1942)	Catching Fireflies (D) https://honolulumuseum.org/collections/6046/	W	ca.1900	HM
100	Masanari, Shiomi	Case (Inrō) with Design of Fireflies […] (A) https://www.metmuseum.org/art/collection/search/58837	O	19th c.	MET
101	Masao, Maeda (1904–1974)	The Tale of Genji – Hotaru (L, G) https://ukiyo-e.org/image/artelino/39361g1	W	ca.1950s	UKIYO
102	Matichiro, Isoda (1907–1998)	Autumn Moon of Higashiyama (Moonrise over Higashiyama) (D) https://ukiyo-e.org/image/jaodb/Isoda_Mataichiro-No_Series-Autumn_Moon_of_Higashiyama_Moonrise_over_Yoneyama-00033268-040322-F06	W	1952	UKIYO
103	Mitsuharu (1770–1838)	Pipe Case with Firefly Motif (A) https://collections.artsmia.org/art/139919/pipe-case-with-firefly-motif-mitsuharu	O	1840s	MIA
104	Mitsuhiro, Ohara (1810–1875)	Firefly on a Pepper (netsuke) (A) http://asianart.emuseum.com/view/objects/asitem/items$0040:8432	O	1825–1875	AAM
105	Mitsuhiro, Ohara (1810–1875)	Firefly on a Paper Bag (netsuke) (A) http://asianart.emuseum.com/view/objects/asitem/items$0040:8433	O	1830–1875	AAM
106	Nakahara, Juni’chi (1913–1983)	Firefly Cage (from Postcards of Japanese Maidens series) (S, G)	L	1930s	.
107	Nishikawa Sukenobu (1671–1751)	Fireflies (from the book Ehon Makuzugahara) (L) https://collections.mfa.org/objects/326804	W	1759	MFAB
108	Nobukazu, Watanabe (Yosai) (1872–1944)	Love Of Fireflies (S, G) https://ukiyo-e.org/image/ohmi/Nobukazu_Yosai-24_Favourites_Of_Beautiful_ Ladies-Love_Of_Fireflies-01-02-25-2007-8331-x2000	L	1896	UKIYO
109	Nobukazu, Watanabe (Yosai) (1872–1944)	Beauties Strolling in an Evening Garden Viewing Fireflies (S, G) https://ukiyo-e.org/image/jaodb/Nobukazu_Yosai-No_Series-Beauties_strolling_ in_an_evening_garden_viewing_fireflies-00043295-111026-F12	W, T	1890	UKIYO
110	Ogawa, Kazuma (Kazumasa)	Women Hunting for Fireflies (Z) http://oldphoto.lb.nagasaki-u.ac.jp/global/search/jp_detail.php?id=1879	PH	unk.	NUL
111	Raisho, Nakajima (1796-1871)	Cricket in a Cage (X) https://www.britishmuseum.org/collection/object/A_1980-1022-0-43	W	1796–1871	BRM
112	Sadahide, Utagawa (1807-1878)	Eastern Brocade, Picture of Stylish Firefly Catching (L) [34], pp. 144–145	W, T	1820s	BOOK
113	Seibei, Kajima (Kashima)	Girls Holding Insect Cages (Z, FN) http://oldphoto.lb.nagasaki-u.ac.jp/global/search/jp_detail.php?id=4328	PH	unk.	NUL
114	Seibei, Kajima (Kashima)	An Insect Cage Vender and Girls (Z, FN) http://oldphoto.lb.nagasaki-u.ac.jp/global/search/jp_detail.php?id=4334	PH	unk.	NUL
115	Seihō, Takeuchi (1864–1942)	Bamboo Leaves and Firefly (Z) https://asia.si.edu/object/S2003.8.2487/	W	20th c.	SNMAA
116	Seitei, Watanabe (1851–1918)	July (Firefly, Summer Twilight) (Flowers […] series) (A) https://asia.si.edu/object/F2014.8.45.7/	PA	1851–1918	SNMAA
117	Shigenaga, Nishimura (1697–1756)	Fireflies at the Sahô River (No. 3, Eight Views of Nara series) (L) https://collections.mfa.org/objects/226019	W	ca.1731	MFAB
118	Shinsui, Itô (1898–1972)	Firefly Catching (First Collection of Modern Beauties series) (A, D, G) https://collections.artsmia.org/art/62300/firefly-catching-ito-shinsui	W	1931	MIA
119	Shinsui, Itô (1898–1972)	Firefly (Second Series of Modern Beauties) (L, G) https://collections.mfa.org/objects/253607	W	1934	MFAB
120	Shinsui, Itô (1898–1972)	Firefly (A, G) https://www.fujiarts.com/cgi-bin/item.pl?item=964360	W	1898–1972	FUJI
121	Shiun, Kondō (active ca.1915–1940)	Firefly Romance (S, G) https://ukiyo-e.org/image/jaodb/Kondo_Shiun-No_Series-Firefly_Romance-00041658-081027-F12	L	ca.1920s	UKIYO
122	Shôen, Uemura (1875–1949)	Firefly (L, G) https://www.fujiarts.com/cgi-bin/item.pl?item=964358	W	1875–1949	FUJI
123	Shôen, Uemura (1875–1949)	Firefly (S, G) https://artsandculture.google.com/asset/firefly/4gG0KvWQNZajUQ	PA	1913	GAC
124	Shôen, Uemura (1875–1949)	Catching Fireflies (D) https://artsandculture.google.com/asset/catching-fireflies/BgFAryQksI3y9w	PA	1932	GAC
125	Shôen, Uemura (1875–1949)	Evening in the Early Summer (L, G) https://ukiyo-e.org/image/artelino/38317g1	W	1940–1950s	UKIYO
126	Shōnen, Suzuki (1849–1918)	Fireflies at Uji River (A, G) https://collections.artsmia.org/art/118428/fireflies-at-uji-river-suzuki-shonen	PA	1849–1918	MIA
127	Shōnen, Suzuki (1849–1918)	Fireflies Over the Uji River by Moonlight (A, G) https://www.metmuseum.org/art/collection/search/57166	PA	1849–1918	MET
128	Shosai, Ikkei (active ca.1860-1870s)	Thirty-six Amusing Views of Famous Places in Tokyo: Negishi Village (S, G) https://museumcollection.tokyo/works/6232040/	W	1872	TM
129	Shōsō, Mishima (1856–1928)	Watching Fireflies on a Summer Night (D) https://www.metmuseum.org/art/collection/search/55970	W	1856–1928	MET
130	Shōtei (Hiroaki), Takahashi (1871–1944)	Catching Fireflies (D) https://asia.si.edu/object/S2003.8.2358/	W	1909–1923	SNMAA
131	Shōtei (Hiroaki), Takahashi (1871–1944)	Insect Vendor, Summer Evening (D, FN) https://ukiyo-e.org/image/artelino/42048g1	W	ca.1910–1930s	UKIYO
132	Shōtei (Hiroaki), Takahashi (1871–1944)	Moon over Sekiguchi (D) https://ukiyo-e.org/image/artelino/48934g1	W	ca.1930s	UKIYO
133	Shōtei (Hiroaki), Takahashi (1871–1944)	Firefly Hunting in Cool Breeze (D) http://www.jaodb.com/db/ItemDetail.asp?item=33643	W	ca.1930s	UKIYO
134	Shōtei (Hiroaki), Takahashi (1871–1944)	Nightscape with Fireflies (D) https://asia.si.edu/object/S2003.8.2409/	W	1871–1944	SNMAA
135	Shōun, Yamamoto (1870–1965)	The Fireflies, Imasugata (S, G) https://ukiyo-e.org/image/jaodb/Yamamoto_Shoun-Fashions_of_Today-The_fireflies_Imasugata-00035242-031030-F06	W	ca.1906	UKIYO
136	Shōun, Yamamoto (1870–1965)	Landscape (D) https://asia.si.edu/object/S2003.8.3381/	W	1900s	SNMAA
137	Shuncho, Katsukawa (active ca.1780–1801)	Vendor of Singing Insects at Shinobazu Pond (Z, FN) https://collections.mfa.org/objects/254959	W	1780s	MFAB
138	Shunchösai, Takehara (?–1801)	Firefly Hunting and Thunderbolt (Z) https://honolulumuseum.org/collections/45274/	W	1797	HM
139	Shunman, Kubo (1757–1820)	Beauty with Fireflies (D) https://www.clevelandart.org/art/2015.85	PA	early 1800s	CMA
140	Shunsen (Shunkô II), Katsukawa (1762–1830)	Women and Children Catching Fireflies (S) https://collections.mfa.org/objects/217074	W	1762–1830	MFAB
141	Shunsen (Shunkô II), Katsukawa (1762–1830)	Catching Fireflies (S) https://www.loc.gov/pictures/collection/jpd/item/2009615584/	W	ca.1830	LOC
142	Shuntei, Miyagawa (1873–1914)	Pleasures of the World: Catching Fireflies (L, G) https://ukiyo-e.org/image/scholten/10-2900w	W	1898	UKIYO
143	Shuntei, Miyagawa (1873–1914)	Firefly Hunting (D) https://ukiyo-e.org/image/artelino/27418g1	W	ca.1900–1910	UKIYO
144	Shunzan, Katsukawa (active ca.1781–1801)	Ladies Catching Fireflies (L) https://art.nelson-atkins.org/objects/26409	W, T-l	1781–1801	NAM
145	Shunzan, Katsukawa (active ca.1781–1801)	Ladies Catching Fireflies (L) https://art.nelson-atkins.org/objects/6847	W, T-c	1781–1801	NAM
146	Shunzan, Katsukawa (active ca.1781–1801)	Ladies Catching Fireflies (L) https://art.nelson-atkins.org/objects/1953	W, T-r	1781–1801	NAM
147	Sômin V, Yokoya (1795–?)	Shain Studying by the Light of a Bag of Fireflies (kozuka) (S) https://collections.mfa.org/objects/18654	O	mid-19th c.	MFAB
148	Sōzan, Itō (1884–?, active 1919–1926)	Fireflies At Night (A, G) https://asia.si.edu/object/S2003.8.351/	W	pre-1923	SNMAA
149	Sōzan, Itō (1884–?, active 1919–1926)	Fireflies on Grasses at Night (A, G) https://collections.mfa.org/objects/321526	W	1920s	MFAB
150	Tadamasa, Ueno (1904–1970)	Firefly - Kabuki Calendar (S, G) https://ukiyo-e.org/image/artelino/25286g1	W	ca.1950s	UKIYO
151	Takeji, Asano (1900–1999)	Fireflies at Byodo-In Temple ([…] Temples in Kyoto series) D) https://ukiyo-e.org/image/jaodb/Asano_Takeji-Snow_Moon_and_Flowers_at_ Temples_in_Kyoto-Fireflies_at_Byodo_In_Temple-00029439-041002-F12	W	1941	UKIYO
152	Tanaka, Taisuke	Man Collecting Insects (Z, NP) https://www.edohakuarchives.jp/detail-5485.html	PH	1911–1920	TM
153	Tetsunao	Insect-Cage Incense Burner (N) https://www.artic.edu/artworks/193244/insect-cage-incense-burner	O	1875-1925	AIC
154	Toju	Boats on a Lake with People Catching Fireflies (inro) (L, G) https://www.britishmuseum.org/collection/object/A_1981-0203-26-a	O	19th c.	BRM
155	Tokuriki, Tomikichiro (1902–2000)	Fireflies and the Uji River (15 Views of Kyoto series) (D) https://ukiyo-e.org/image/jaodb/Tokuriki_Tomikichiro-15_Views_of_Kyoto-Fireflies_and_the_Uji_River-00033958-040622-F06	W	1930s–1950s	UKIYO
156	Toshihide, Migita (1863–1925)	Satsuki (Fifth Month) (Twelve Forms of Beauty series) (D) https://www.britishmuseum.org/collection/object/A_1906-1220-0-1520	W	1901	BRM
157	Toshikata, Mizuno (1866–1908)	Chasing Fireflies, A Lady of the Tenmei Era ([…] series) (D) https://www.clevelandart.org/art/2017.73	W	1894	CMA
158	Toyohiro, Utagawa (1773–1828)	Women Chasing Fireflies (S) https://collections.mfa.org/objects/26600	PA	1793–1794	MFAB
159	Toyonobu, Ishikawa (1711–1785)	Beauty Holding a Firefly Cage (L) https://collections.artsmia.org/art/8604	PA	mid-18th c.	MIA
160	Tsunemasa, Kawamata (active 1716–1748)	Two Girls Catching Fireflies (L) https://www.metmuseum.org/art/collection/search/45770	PA	1716–1748	MET
161	Unknown artist	Bamboo (A, G) https://asia.si.edu/object/S2003.8.3692/	W	20th c.	SNMAA
162	Unknown artist	Fireflies (L, G) https://ukiyo-e.org/image/artelino/21698g1	W	unk.	UKIYO
163	Unknown artist	Box for Inkstone and Writing Implements (Suzuribako) (A, G) https://www.metmuseum.org/art/collection/search/58120	O	19th c.	MET
164	Unknown artist	Catching Firefly (Z) https://harvardartmuseums.org/collections/object/71250	PH	1870–1880s	HAM
165	Unknown artist	Fire-fly Cage (Z) https://www.metmuseum.org/art/collection/search/48814	O	1840	MET
166	Unknown artist	Fireflies (A) https://collections.mfa.org/objects/428346	PA	unk.	MFAB
167	Unknown artist	Fireflies and Yago (A) https://ukiyo-e.org/image/japancoll/p225--fireflies-and-yago-8862	W	unk.	UKIYO
168	Unknown artist	Fireflies on Stalks (A) https://collections.mfa.org/objects/428187	PA	unk.	MFAB
169	Unknown artist	Firefly and Moon (A) https://ukiyo-e.org/image/artelino/52815g1	W	unk.	UKIYO
170	Unknown artist	Firefly and Spider Web (A, G) https://collections.mfa.org/objects/394552	W	1868–1912	MFAB
171	Unknown artist	Firefly Viewing Party (S) https://collections.artsmia.org/art/42489/firefly-viewing-party-unknown-japanese	W	ca.1818–1829	MIA
172	Unknown artist	Hotarujaya and Ichinose-bashi Bridge (Otaru Tea House at Nagasaki) (Z) http://oldphoto.lb.nagasaki-u.ac.jp/global/search/jp_detail.php?id=5635	PH	unk.	NUL
173	Unknown artist	Incense Box with Autumn Grasses and Insect Cage (Z, NP) https://www.metmuseum.org/art/collection/search/57700	O	mid-19th c.	MET
174	Unknown artist	Lantern and Firefly (A) https://ukiyo-e.org/image/artelino/36715g1	W	unk.	UKIYO
175	Unknown artist	Sake Cups with Maki-e Design of Oki-no-ishi (L) https://artsandculture.google.com/asset/set-of-sake-cups-with-maki-e-design-of-oki-no-ishi-unknown/TAECi9Y9uJfmvQ	O	18th–19th c.	GAC
176	Unknown artist	Small Box in the Shape of a Firefly Cage (S, G) https://collections.artsmia.org/art/455	O	19th c.	MIA
177	Unknown artist	Small Box (A, G) https://collections.vam.ac.uk/item/O487187/small-box-unknown/	O	unk.	VAM
178	Unknown artist	Summer Robe (Katabira) with Autumn Flowers and Insect Cages (Z, NP) https://www.metmuseum.org/art/collection/search/785518	O	ca.early 19th c.	MET
179	Unknown artist	Unlined Summer Kimono […] (Z, NP) https://www.metmuseum.org/art/collection/search/50807	O	early 20th c.	MET
180	Unknown artist	Woman Hunting Fireflies, Man Falling from Bench (Z) https://collections.mfa.org/objects/540671	W	1600–1868	MFAB
181	Unknown artist	Writing Box (L) https://collections.vam.ac.uk/item/O16125/writing-box/	O	early 19th c.	VAM
182	Unknown artist	Young Women on a Summer Evening (S) https://collections.mfa.org/objects/256194	W, D?	1671–1750	MFAB
183	Utamaro, Kitagawa (1753–1806)	Tree cricket; Firefly, from the Picture Book of Crawling Creatures (A, G) https://www.metmuseum.org/art/collection/search/37288	W	1788	MET
184	Utamaro, Kitagawa (1753–1806)	Beauty and an Insect Cage (Z) https://ukiyo-e.org/image/ritsumei/Z0168-106	W	1790	UKIYO
185	Utamaro, Kitagawa (1753–1806)	Three Women Seated […] with Three Children and Cat Playing (Z) https://www.britishmuseum.org/collection/object/A_1912-0416-0-220	W, T	1794–1795	BRM
186	Utamaro, Kitagawa (1753–1806)	Catching fireflies (Hotaru gari) (L) https://www.metmuseum.org/art/collection/search/36630	W, T	ca.1796–1797	MET
187	Utamaro, Kitagawa (1753–1806)	Mother and Children Enjoying Fireflies (N) https://harvardartmuseums.org/collections/object/210591	W	1753–1806	HAM
188	Utamaro, Kitagawa (1753–1806)	Picture of the Upper Class, Three Ranks of Young Women […] (NP) https://collections.mfa.org/objects/234042	W	ca.1794–1795	MFAB
189	Utamaro, Kitagawa (1753–1806)	The Insect Vendor (Z, FN) https://www.fujiarts.com/cgi-bin/item.pl?item=930918	W	unk.	FUJI
190	Yamamoto, Shōkoku (1870–1965)	Firefly Cage (D) https://www.loc.gov/pictures/collection/jpd/item/2008660365/	W	1900–1965	LOC
191	Yasutada, Koma	Case (Inrō) with Design of Clamshells and Fireflies (A, G) https://www.metmuseum.org/art/collection/search/54242	O	late 18th c.	MET
192	Yoshio, Okado (1977–)	Fireflies, Chapter 25 (A, G) https://honolulumuseum.org/collections/52801/	W	ca.1978	HM
193	Yoshitoshi, Tsukioka (1839–1892)	Thirty-two Aspects of Women: Delighted […] (L, G) https://ukiyo-e.org/image/scholten/10-3207w	W	1888	UKIYO
194	Yoshitsuya, Utagawa (1822–1866)	The Actor Kawarazaki Gonjūrō Surrounded by Fireflies (L, G) https://collections.lacma.org/node/213481	W	1862	LACMA
195	Yurimoto, Keiko (1906–?)	Catching Fireflies - Life of Japanese Children (D) https://ukiyo-e.org/image/jaodb/Yurimoto_Keiko-No_Series-Untitled_Catching_Fireflies-00034209-030511-F06	W	ca.1950	UKIYO
196	Yurimoto, Keiko (1906–?)	Catching Fireflies - Life of Japanese Children (different colors) (D) https://ukiyo-e.org/image/jaodb/Yurimoto_Keiko-The_Life_Of_Japanese_Children-Postcard_Catching_Fireflies-00032962-021026-F06	W	1950s	UKIYO
197	Yushin, Ayaoka (1846–1910)	Fireflies (A, G) https://www.britishmuseum.org/collection/object/A_1981-0609-0-16	W	1880–1886	BRM
198	Zeshin, Shibata (1807–1891)	Fan and Insect (A) https://honolulumuseum.org/collections/54920/	PA	ca.1833–1872	HM
199	Zeshin, Shibata (1807–1891)	Rice Stalks and Fireflies (A) https://asia.si.edu/object/S1996.89/	W	1870–1879	SNMAA
200	Zeshin, Shibata (1807–1891)	A Firefly on Grasses (A) https://honolulumuseum.org/collections/8495/	W	ca.1880s	HM
201	Zeshin, Shibata (1807–1891)	Firefly Catching (S) https://museumcollection.tokyo/en/works/6234133/	W	1888	TM
202	Zeshin, Shibata (1807–1891)	Firefly on Flowering Plant and Bamboo Sieve on Lacquered Tray (A) https://asia.si.edu/object/S2003.8.2165/	W	1833–1891	SNMAA
203	Zeshin, Shibata (1807–1891)	Firefly and Grasses with Windshade (A, G) https://www.britishmuseum.org/collection/object/A_1928-0720-0-42	PA	1850–1891	BRM
204	Zeshin, Shibata (1807–1891)	Insects Seller (NP) https://museumcollection.tokyo/en/works/6234394/	W	19th c.	TM
205	Zeshin, Shibata (1807–1891)	Fan and Insect Cage (Z, NP) https://www.metmuseum.org/art/collection/search/57202	PA	1807–1891	MET

^1^ = A = relatively accurate representation of firefly, L = less accurate and more generalized firefly, S = more symbolic or abstract firefly, D = firefly represented by yellowish/greenish dots, G = firefly has yellowish/greenish abdomen or yellowish /greenish dots representing bioluminescence, Z = insects were not shown or picture quality too poor, FN = artwork likely related to fireflies and other insects, NP = context indicated fireflies (or context unclear) but artwork is likely not related to fireflies, N = context indicated fireflies (or context unclear) but artwork is definitely not related to fireflies, X = context indicated artwork is not related to fireflies. ^2^ = L = lithograph, O = object, PA = painting, PH = photo, W = woodblock print (D = diptych, P = pentaptych, R = pair of six-fold screens, T = triptych, T-l = left panel of triptych, T-c = center panel of triptych, T-r = right panel of triptych, TE = tetraptych). ^3^ = AAM: Asian Art Museum, San Francisco, CA, USA, asianart.emuseum.com; AIC: Art Institute of Chicago, IL, USA, www.artic.edu; BOOK: published book; BRM: The British Museum, London, England, www.britishmuseum.org; CMA: The Cleveland Museum of Art, OH, USA, www.clevelandart.org; FUJI: Fuji Arts dealer website, www.fujiarts.com; GAC: Google Arts & Culture, artsandculture.google.com; HAM: Harvard Art Museums, Boston, MA, USA, harvardartmuseums.org; HM: The Honolulu Museum of Art, HI USA, honolulumuseum.org; LACMA: Los Angeles County Museum of Art, CA, USA, www.lacma.org; LOC: The Library of Congress, Washington, DC, USA, www.log.gov; MET: The Metropolitan Museum of Art, New York, NY, USA, www.metmuseum.org; MFAB: Museum of Fine Arts Boston, MA USA, www.mfa.org; MFAH: Museum of Fine Arts Houston, TX, USA, www.mfah.org; MIA: Minneapolis Institute of Art, MN, USA, new.artsmia.org; NAM: Nelson Atkins Museum of Art, Kansas City, MO, USA, nelson-atkins.org; NUL: Nagasaki University Library, Nagasaki City, Japan, nagasaki-u.ac.jp; SNMAA: Smithsonian National Museum of Asian Art, Washington, DC, USA, asia.si.edu; SOT: Sotheby’s dealer website, www.sothebys.com; TM: Edo-Tokyo Museum Digital Archives, Tokyo, Japan, www.edohakuarchives.jp; UKIYO: Ukiyo-e.org database; VAM: Victoria & Albert Museum, London, England, www.vam.ac.uk. (Websites were accessed multiple times from 4 January 2022 to 22 August 2022).
